# Short Exposure to Ethanol Diminishes Caspase-1 and ASC Activation in Human HepG2 Cells In Vitro

**DOI:** 10.3390/ijms21093196

**Published:** 2020-04-30

**Authors:** Jason-Alexander Hörauf, Shinwan Kany, Andrea Janicova, Baolin Xu, Teodora Vrdoljak, Ramona Sturm, Ildiko Rita Dunay, Lukas Martin, Borna Relja

**Affiliations:** 1 Department of Trauma, Hand and Reconstructive Surgery, Goethe University Frankfurt, 60438 Frankfurt am Main, Germany; Jason-Alexander.Hoerauf@kgu.de (J.-A.H.); Ramona.Sturm@kgu.de (R.S.); 2 Experimental Radiology, Department of Radiology and Nuclear Medicine, Otto von Guericke University Magdeburg, 39108 Magdeburg, Germany; s.kany@uke.de (S.K.); Andrea.Janicova@med.ovgu.de (A.J.); xubaolin325@outlook.com (B.X.); 3 Department of Cardiology with Emphasis on Electrophysiology, University Heart Centre, University Hospital Hamburg-Eppendorf, 20251 Hamburg, Germany; 4 Department of Diagnostic and Interventional Radiology, University Hospital Dubrava, University of Zagreb School of Medicine, HR-10000 Zagreb, Croatia; tea.vrdoljak@gmail.com; 5 Institute of Inflammation and Neurodegeneration, Otto von Guericke University Magdeburg, 39108 Magdeburg, Germany; ildiko.dunay@med.ovgu.de; 6 Department of Intensive Care and Intermediate Care, University Hospital RWTH Aachen, 52074 Aachen, Germany; lmartin@ukaachen.de

**Keywords:** inflammation, inflammasome, caspase-1, alcohol, liver, in vitro

## Abstract

This paper discusses how the assembly of pro-caspase-1 and apoptosis-associated speck-like protein containing a caspase-recruitment domain (ASC) in macromolecular protein complexes, inflammasomes, activates caspase-1. The present study investigates the molecular mechanisms of inflammasome activation in HepG2 cells and examines how short exposures to ethanol (EtOH) affect inflammasome activation. HepG2 cells were treated with lipopolysaccharide (LPS), ATP or nigericin (NIG) in a two-step model. After LPS priming, ATP or NIG were added. As inhibitors, sodium orthovanadate (general inhibitor of tyrosine phosphatases), AC-YVAD-CMK (caspase-1 inhibitor) or AZ10606120 (purinergic receptor P2X7R inhibitor) were applied after LPS priming. To monitor the inflammasome activation, the caspase-1 activity, ASC speck formation, reactive oxygen species (ROS) production and cell death were analyzed. To elucidate the mechanistical approach of EtOH to the inflammasome assembly, the cells were treated with EtOH either under simultaneous LPS administration or concurrently with ATP or NIG application. The co-stimulation with LPS and ATP induced a significant ASC speck formation, caspase-1 activation, cell death and ROS generation. The inhibition of the ATP-dependent purinoreceptor P2X7 decreased the caspase-1 activation, whereas sodium orthovanadate significantly induced caspase-1. Additional treatment with EtOH reversed the LPS and ATP-induced caspase-1 activation, ASC speck formation and ROS production. The ASC speck formation and caspase-1 induction require a two-step signaling with LPS and ATP in HepG2 cells. Inflammasome activation may depend on P2X7. The molecular pathway of an acute effect of EtOH on inflammasomes may involve a reduction in ROS generation, which in turn may increase the activity of tyrosine phosphatases.

## 1. Introduction

Inflammasomes, cytosolic multi-protein complexes, are of central importance to inflammatory processes as they promote the maturation of certain pro-inflammatory cytokines, notably interleukin (IL)-1β or IL-18 [1]. A typical multimeric inflammasome consists of an adaptor protein or apoptosis-associated speck-like protein containing a caspase recruitment domain (CARD) (ASC), a pro-caspase-1 and a cytosolic pattern recognition receptor (PRR), which is capable of detecting intracellular pathogen-associated molecular patterns (PAMP)—related to infectious pathogens—and/or damage-associated molecular patterns (DAMP) that are related to cell stress [2]. 

Gene mutations of, for example, NLRP3 are playing a central role in the pathogenesis of several auto-inflammatory diseases such as cryopyrin-associated periodic syndrome (CAPS), including familial cold autoinflammatory syndrome (FCAS) or Muckle–Wells syndrome (MWS) [3]. Recent data have indicated that NLRP3 was also involved in the development of globally widespread diseases like diabetes type 2 and Alzheimer’s disease [4,5]. NLRP3 is characterized by three structural domains: (i) a pyrin domain (PYD), (ii) a nucleotide-binding domain (NBD or NACHT) and (iii) a leucine-rich repeat domain (LRR), which is considered to play a pivotal role in the recognition of DAMP and/or PAMP [6]. Activation of the NLRP3 inflammasome by macrophages requires two successive steps, which involve a first so-called priming step (step 1) prior to a second NLRP3-specific activating signal (step 2) [7]. A common trigger for step 1 is, for example, the activation of the PRR toll-like receptor (TLR)4 by lipopolysaccharide (LPS), or the pro-inflammatory cytokine tumor necrosis factor alpha (TNF-α) activating the transcription factor NF-κB and thereby notably upregulating pro-IL-1β [7]. After priming NLRP3, a second “activating” step is required to form the functional inflammasome complex containing ASC and pro-caspase-1. Numerous inducers of the second signal have been discovered; among those are exogenous PAMP and endogenous DAMP such as bacterial toxins, nigericin, adenosine triphosphate (ATP), gout-associated crystals (monosodium urate) or calcium pyrophosphate dihydrate crystals [8,9]. Due to the large variety of potential activators, it seems rather unlikely that NLRP3 binds and interacts directly or specifically with all these agents. Three molecular mechanisms of NLRP3 activation which potentially converge those physically and chemically diverse stimuli into a general cellular signal have primarily been discussed. These mechanisms comprise potassium (K^+^) efflux mediated via the purinergic receptor P2RX7 [10], lysosomal destabilization and rupture [11] and the generation of reactive oxygen species (ROS) [12]. 

After sensing step 1 and step 2, the NLRP3 inflammasome assembly is mediated by a PYD/PYD interaction between ASC and NLRP3 and a CARD/CARD interaction between ASC and pro-caspase-1, resulting in so-called ASC-formed cytosolic specks [6]. This speck formation promotes the auto-activation of inactive pro-caspase-1 into its active form, caspase-1 [6,13]. In consequence, active caspase-1 proteolytically cleaves the precursor of, for example, IL-1β into its mature active form, which is secreted [6,14]. Caspase-1 is the leading enzyme to mediate a highly inflammatory form of the programmed cell death, so-called pyroptosis, which is characterized by rapid cell lysis and the release of pro-inflammatory cytokines [2,15].

Besides its preferred side-effects, such as the reduction in anxiety or relaxation, alcohol consumption is harmful for many organ systems, e.g., the pancreas, liver and immune system [16]. Notably, its impact on the innate immune system appears to be strongly dependent on drinking behavior. While acute alcohol consumption mediates rather anti-inflammatory effects by suppressing pro-inflammatory cytokines [17,18], chronic alcohol use induces pro-inflammatory changes and increases the susceptibility to viral and bacterial infections [19].

Inflammasomes seem to play a central role in alterations to the innate immune system that are mediated by alcohol. Chronic exposure to alcohol amplifies the neuro-inflammation mediated by NLRP3 activation in both astrocytes and glia cells [20] and results in the NLRP3-mediated inflammation in the pancreas of albino wistar rats [21]. Wree et al. demonstrated a central role of NLRP3 inflammasome activation in severe liver inflammation accompanied by increased neutrophil infiltration, enhanced hepatocyte pyroptosis and the induction of hepatic stellate cell (HSC) activation leading to collagen deposition and the development of fibrosis [22]. Upon chronic ethanol exposure, the upregulation of pro-inflammatory cytokines, such as IL-1β or TNF-α, was observed in Kupffer cells and liver-resident macrophages, resulting in hepatocyte dysfunction and apoptosis [23]. Animals which were chronically fed with alcohol showed the enhanced release and signaling response of IL-1β, caused by an increased activation of NF-κB in hepatocytes [24]. The blocking of the IL-1β signaling markedly attenuated the alcohol-induced liver inflammation, steatosis and damage in a chronic ethanol in vivo model [25].

In contrast, acute or short exposure to alcohol attenuated the NLRP3 inflammasome activation. Nurmi et al. revealed a decreased IL-1β secretion of human macrophages after their acute/short exposure to ethanol by inhibiting caspase-1 activation and the oligomerization of ASC [26]. Hoyt et al. demonstrated that an acute/short exposure of human peripheral blood mononuclear cells (PBMC) to ethanol inhibited ROS production, and that the NLRP3 inflammasome activation is the underlying mechanism [27]. The authors demonstrated that decreased levels of global tyrosine phosphorylation resulted in the attenuated phosphorylation of ASC and thus in significantly less inflammasome activity [27]. 

In previous studies, our group has demonstrated that acute/short exposure to ethanol attenuated the release of pro-inflammatory cytokines in vitro [28,29]. Recently, we figured out that acute/short exposure to ethanol decreased the inflammatory response by inhibiting the canonical pathway of NF-kB signaling in human lung epithelial cells in vitro [28], which also plays a central role in step 1 signaling of inflammasome activation [7]. The impact of the acute exposure of hepatic cells to ethanol is poorly investigated, and nothing is known about the possible interplay with inflammasomes. Thus, this study examines how acute and short exposure to ethanol affects the inflammasome activation in human-derived liver cells in vitro.

## 2. Results

### 2.1. Inflammasome Activation upon Ethanol Administration in Hepato-Derived Cells

#### 2.1.1. LPS and ATP Induce ASC Speck Formation and Activate Caspase-1 in HepG2 Cells

**Figure 1:**
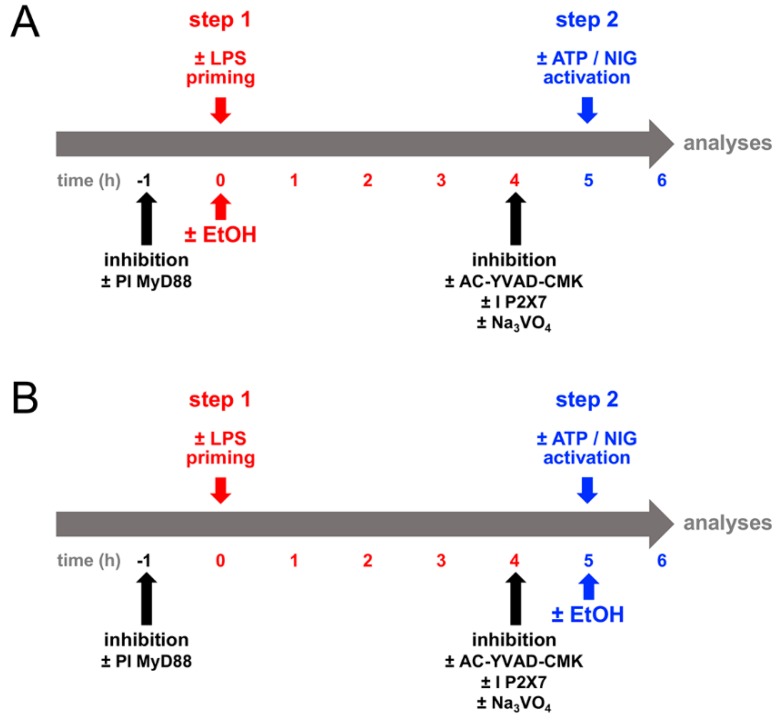
Schematic timeline of the experimental design. (**A**) Human liver-derived HepG2 cells were primed with lipopolysaccharide (LPS, 1 µg/mL, step 1, in red) with simultaneous application of ethanol (EtOH, 170 mM, EtOH 1) for five hours and subsequently treated with ammonium salt (BzATP, ATP, 100 µM) or nigericin (NIG, 50 µM, step 2, in blue) for one hour. In order to elucidate the general principles of inflammasome activation, MyD88 inhibitory peptide (PI MyD88, 25 µM) was applied one hour before LPS priming or sodium orthovanadate (Na_3_VO_4_, 1000 µM), AC-YVAD-CMK (100 µM) and AZ10606120 (I P2X7, 1 µM) were added four hours after LPS priming. (**B**) LPS priming. ATP or NIG treatment as well as the application of the inhibitors were implemented as described in (**A**). EtOH was applied concurrently with ATP or NIG (EtOH 2). Subsequently, all samples were analyzed.

**Figure 2:**
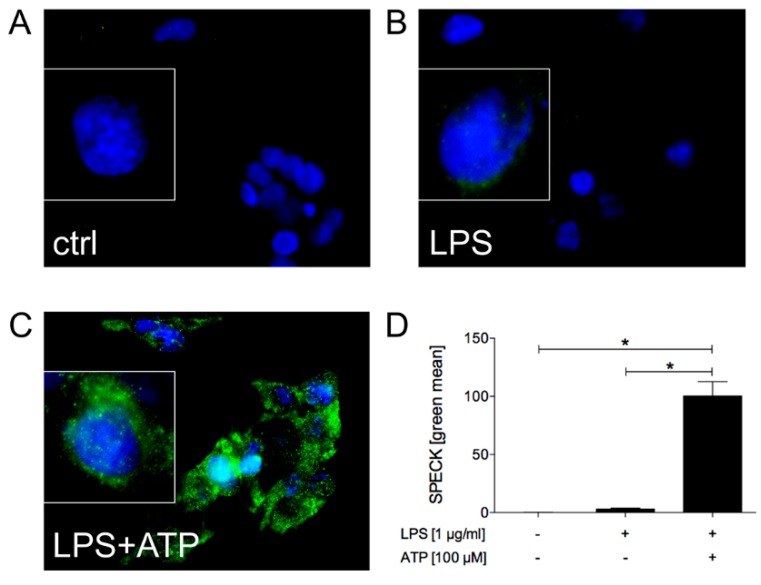
Caspase-recruitment domain (ASC)) speck formation upon stimulation in HepG2. Cells were exposed to a medium (**A**), treated either with lipopolysaccharide (LPS, 1 µg/mL, B) or with LPS and adenosine triphosphate (ATP, 100 µM, C). The co-stimulation with LPS and ATP significantly induced ASC speck formation compared to the control group and single stimulation group with LPS only. The representative immune cytological staining of the ASC speck formation was assessed by fluorescence microscopy (**A**–**C**) and quantified, as described in the materials and methods section (**D**). The mean and standard error of the mean are depicted. *: *p* < 0.05 between the indicated groups.

In order to investigate the inflammasome system in HepG2 cells, we used the most common activation triggers, LPS (first signal, “priming”), ATP and nigericin (second signal, “activation”), which were proven to induce the inflammasome complex in immune cells including macrophages or blood monocytes. Therefore, the HepG2 cells were primed in a first step with LPS, and then ATP was additionally added (Figure 1). Then, the assembly of the ASC speck, which is suitable for monitoring inflammasome activation, was analyzed. The HepG2 cells which were exposed to both LPS and ATP showed significantly higher amounts of fluorescence intensity, uncovering ASC speck formation in comparison to unstimulated cells or cells that were stimulated with LPS only (Figure 2). Summarized, this data indicates that the inflammasome formation in HepG2 cells requires both signals, LPS and ATP.

**Figure 3:**
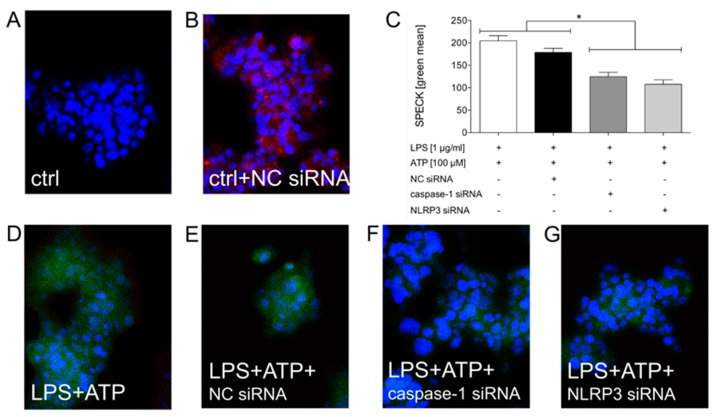
Knock down of the ASC speck formation upon transfection with small interfering RNA (siRNA), directed against caspase-1 or NLRP3 in HepG2. The cells were exposed to the medium (**A**) or transfected with the control small interfering RNA (siRNA) (Silencer™ Cy™3-labeled negative control, NC, (**B**,**E**)). The representative detection of the Cy3-positive cells was assessed by fluorescence microscopy (**B**). Then, the cells were transfected with Cy™3-labeled negative control siRNA (**C**,**D**), caspase-1 siRNA (**C**,**F**) or NLRP3 siRNA (**C**,**G**). Subsequently, the cells were treated with lipopolysaccharide (LPS, 1 µg/mL) and adenosine triphosphate (ATP, 100 µM, C-G). The LPS and ATP-induced ASC speck formation was abolished in the siRNA transfected cells compared to both control groups. The representative immune cytological staining of the ASC speck formation was assessed by fluorescence microscopy (**D**–**G**) and quantified as described in the materials and methods section (**C**). The mean and standard error of the mean are depicted. *: *p* < 0.05 between the indicated groups.

In order to determine whether mechanistical studies on the inflammasome system are applicable in the HepG2 cell line, a targeted gene silencing of the key inflammasome factors was performed. For this purpose, caspase-1 and NLRP3 were knocked down by small interfering RNA (siRNA). A control siRNA, which was linked to Cy3, was applied as the transfection control (red signal in Figure 3b). According to the positive red signal upon transfection in nearly 100% of the cells, the transfection efficacy was significant (Figure 3b). As shown by the representative SPECK staining, the inflammasome assembly was induced by the LPS and ATP stimulation (Figure 3c,d). Transfection with the control siRNA did not significantly modify the LPS and ATP-induced inflammasome assembly (green staining of SPECKs, Figure 3c–e). Upon the transfection of HepG2 cells with either siRNA against caspase-1 or NLRP3, the SPECK formation was significantly reduced compared to either not-transfected cells or to cells that were transfected with the negative control siRNA (Figure 3c–g). This is demonstrated by the decreased SPECK staining in the transfected cells (green staining in Figure 3f,g), as well as by the quantification of data in Figure 3c. In summary, the mechanism of inflammasome activation by LPS and ATP is valid for HepG2 cells. 

**Figure 4:**
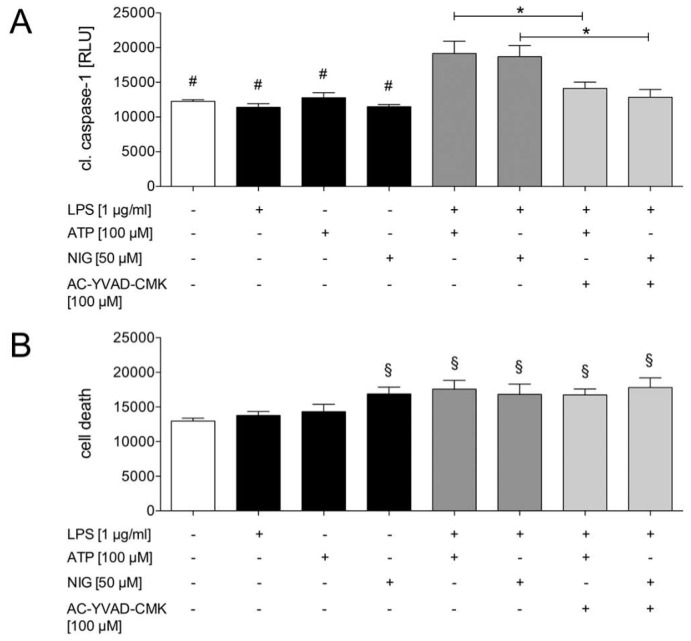
Induction of caspase-1 and cell death in HepG2. Supplementing lipopolysaccharide (LPS, 1 µg/mL), adenosine triphosphate (ATP, 100 µM) or nigericin (NIG, 50 µM) did not activate caspase-1. In contrast, co-stimulation with either LPS and ATP or LPS and NIG significantly induced caspase-1. Additional treatment with the caspase-1 inhibitor AC-YVAD-CMK (100 µM) reduced the caspase-1 activation (**A**). The cells exposed to the medium or to LPS and ATP only showed no induction of cell death. In contrast, exposure to NIG only and co-stimulation with either LPS and ATP or LPS and NIG significantly induced cell death. Additional treatment with the caspase-1 inhibitor AC-YVAD-CMK did not reduce the cell death (**B**). The mean and standard error of the mean are depicted. *p* < 0.05: * versus indicated groups, # versus LPS and ATP and LPS and NIG, and § versus control, LPS or ATP-only group.

Upon inflammasome activation, the zymogen pro-caspase-1 forms a complex with NLRP3, mediated by ASC via its PYD and CARD domains. This assembly auto-activates pro-caspase-1 by autoproteolysis into its active form; that can be detected by the Caspase-Glo^®^ 1 Inflammasome Assay. Treatment with LPS and ATP and LPS and nigericin significantly increased the caspase-1 activity compared to the unstimulated controls and the groups stimulated with LPS, ATP or nigericin only (Figure 4a). Treatment with the AC-YVAD-CMK, a selective irreversible inhibitor of caspase-1, significantly decreased the activity of caspase-1 upon stimulation with LPS and ATP or LPS and nigericin as compared to samples that were not treated with the AC-YVAD-CMK inhibitor but that were stimulated with LPS and ATP or LPS and nigericin (Figure 4a). Subsequently, the inflammasome activation in the HepG2 cells was monitored by active caspase-1, since its activation requires in addition to LPS another signal, such as ATP or nigericin.

The activation of the inflammasome is known to induce a caspase-1-dependent form of cell death, so-called pyroptosis. Therefore, we performed a cell viability assay to examine the influence of the inflammasome activation on the induction of cell death. As expected, exposure to nigericin, LPS and ATP or LPS and nigericin significantly increased the cell death rates compared to unstimulated cells and cells exposed to LPS or ATP only. Inhibiting caspase-1 by adding AC-YVAD-CMK did not show a significant impact on cell death (Figure 4b). In summary, stimulating HepG2 cells with two signals increased the cell death rate.

#### 2.1.2. Caspase-1 Activation in HepG2 Cells is Mediated by the Purinergic P2X7 Receptor

**Figure 5:**
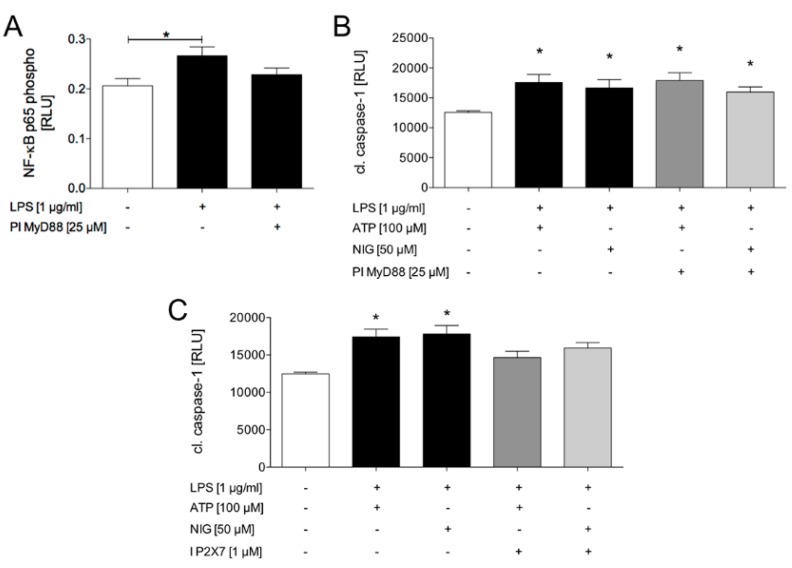
Signal-dependent inflammasome activation in HepG2 cells. The cells exposed to lipopolysaccharide (LPS, 1 µg/mL) showed significantly induced NF-κB p65 phosphorylation. The additional treatment with MyD88 inhibitory peptide (PI MyD88, 25 µM) markedly decreased the LPS-induced NF-kB p65 phosphorylation (**A**). Co-stimulation with either LPS and adenosine triphosphate (ATP, 100 µM) or LPS and nigericin (NIG, 50 µM) significantly induced caspase-1 activation compared to the unstimulated cells. Pre-incubation with MyD88-inhibitory peptide (25 µM) neither reduced the LPS and ATP nor LPS and NIG-induced activation of caspase-1 (**B**). Treatment with the purinergic P2X7 receptor inhibitor (I P2X7, 1 µM) markedly decreased the LPS and ATP and LPS and NIG-induced activation of caspase-1 (**C**). The mean and standard error of the mean are depicted. *p* < 0.05: * versus indicated or control group.

In the next step, we further examined which molecular pathways are involved in the inflammasome activation in HepG2 cells. To investigate whether NF-κB directly participates in caspase-1 activation via the MyD88-dependent pathway, the cells were pre-incubated with the MyD88-inhibitory peptide and subsequently LPS was added. Finally, the phosphorylation of the NF-κB p65 subunit, a reliable marker of NF-κB activation, was determined. LPS induced a significant increase in the phosphorylation of this p65-subunit (Figure 5A). The LPS-induced increase in p65 phosphorylation was markedly but not significantly reduced by the MyD88 inhibitory peptide (Figure 5A). Interestingly, pre-incubation with the MyD88-inhibitory peptide neither affected the LPS and ATP nor LPS and nigericin-induced activation of caspase-1 (Figure 5B). 

Potassium efflux is one of the most discussed mechanisms of inflammasome activation and extracellular ATP was shown to decrease the intracellular K^+^ concentration via binding and activating the purinergic P2X7 receptor. In order to elucidate the influence of ATP on the caspase-1 activation, the HepG2 cells were treated with the P2X7 receptor antagonist AZ10606120 dihydrochloride. The cells which were exposed to LPS and ATP or to LPS and nigericin and simultaneously inhibited with AZ10606120 dihydrochloride showed significantly less active caspase-1 compared to the stimulated cells without the inhibitor (Figure 5C). These data suggest that inflammasome activation, as represented by active caspase-1 in HepG2 cells, is triggered by the purinergic P2X7 receptor.

#### 2.1.3. Ethanol Diminishes Caspase-1 Activity and ASC Speck Formation

**Figure 6:**
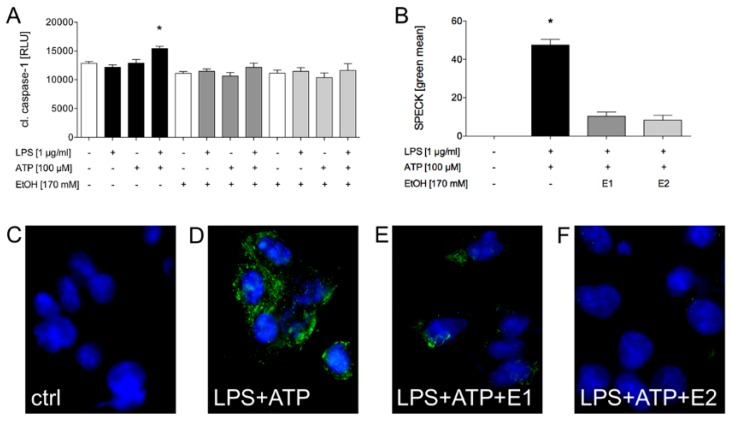
Ethanol impaired the caspase-1 activity and reduced the ASC speck formation. The lipopolysaccharide (LPS, 1 µg/mL) and adenosine triphosphate (ATP, 100 µM)-induced caspase-1 activity was significantly reduced by the ethanol administration (EtOH) at each time point (E1, added simultaneously with LPS or E2, added simultaneously with ATP) (**A**). The co-stimulation with LPS and ATP significantly induced the ASC speck formation compared to the control group. Additional exposure to ethanol, added simultaneously with LPS or with ATP significantly diminished the LPS and ATP-induced ASC speck formation. Representative immune cytological staining of the ASC speck formation was quantified, as described in the materials and methods section (**B**), and assessed by fluorescence microscopy (**C**–**F**). The mean and standard error of the mean are depicted. *p* < 0.05: * versus all.

**Figure 7:**
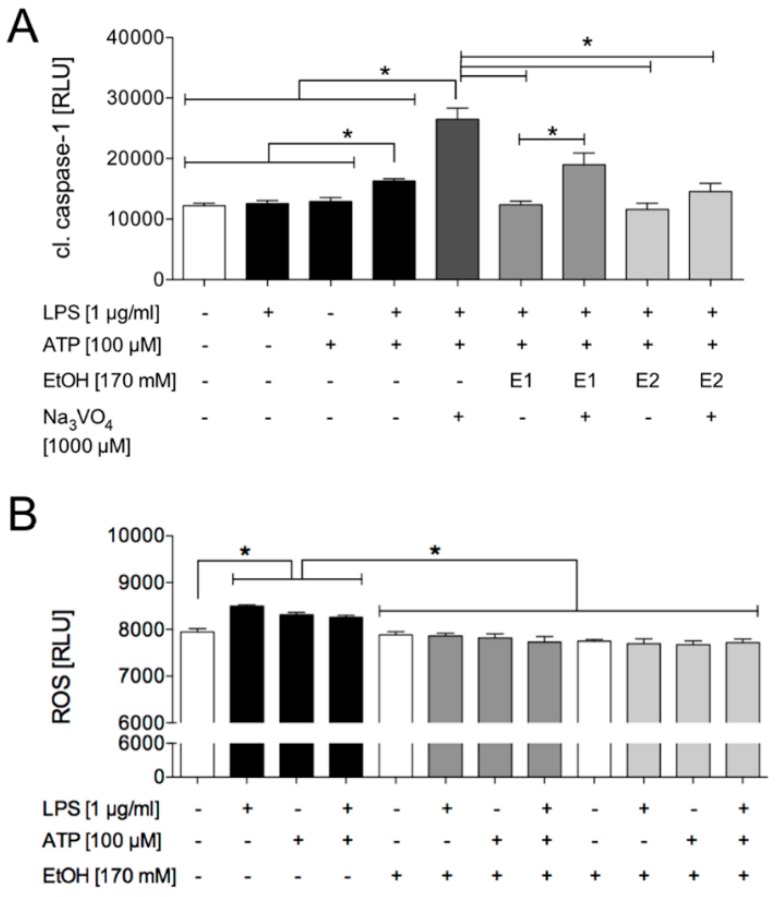
Ethanol lowered the sodium orthovanadate-induced caspase-1 activation and also diminished the reactive oxygen species (ROS) generation. The co-stimulation with lipopolysaccharide (LPS, 1 µg/mL) and adenosine triphosphate (ATP, 100 µM) significantly induced the caspase-1 activation in comparison to the unstimulated control group and single stimulation group with LPS or ATP only. Further treatment with sodium orthovanadate (Na_3_VO_4_, 1000 µM) enhanced the caspase-1 activation additionally. In contrast, exposure to ethanol (EtOH) at the first time point (E1) significantly decreased the caspase-1 activity. Exposure to sodium orthovanadate in the E1-treated cells significantly increased the caspase-1 activity compared to E1 in the LPS and ATP-stimulated cells. (**A**). LPS, ATP and co-stimulation with LPS and ATP significantly induced ROS production. The administration of EtOH at each time point significantly reduced the generation of ROS compared to the stimulated samples (**B**). The mean and standard error of the mean are depicted. *p* < 0.05: *versus indicated.

In order to investigate the influence of ethanol on caspase-1 activation, EtOH was applied to HepG2 cells at two different time points to determine its mechanistical approach. The cells were either treated simultaneously with LPS and EtOH (EtOH1) and subsequently ATP, or the cells were primed with LPS and then simultaneously treated with ATP and EtOH (EtOH2). The LPS and ATP-induced caspase-1 activity was significantly diminished by EtOH, regardless of the time of its application (Figure 6A and Figure 7A). Consistent with these findings, EtOH1 and EtOH2 significantly decreased the LPS and ATP-induced ASC speck formation (Figure 6b–f). This data suggests that EtOH reduced the inflammasome activation. Moreover, its inhibiting effect was present even if it was applied after the first signal (LPS), as represented by active caspase-1 in HepG2 cells, indicating that EtOH may affect the inflammasome formation at the transduction of the second signal.

The administration of sodium orthovanadate, a general inhibitor of protein phosphatases, significantly enhanced the caspase-1 activity (Figure 7a). The additional application of EtOH to sodium orthovanadate at the first time point significantly decreased the caspase-1 activity, but less so than EtOH does in the LPS and ATP group. Similar results were detected at the second time point of the EtOH administration (Figure 7a). This data suggests that the decreased activity of the tyrosine phosphatases supported the inflammasome activation in HepG2 cells. 

#### 2.1.4. LPS + ATP-Induced ROS Generation Was Decreased by Ethanol Administration

Beside potassium efflux, generation of ROS is another widely discussed mechanism of inflammasome activation. In order to investigate the effects of generic inflammasome activators like LPS or ATP on ROS production by HepG2 cells, the cells were treated either with LPS, ATP or LPS and ATP following the experimental protocol. LPS, ATP and LPS and ATP significantly increased the ROS formation compared to the untreated control (Figure 7b). To examine the influence of ethanol on the production of ROS, EtOH was added at two time points as described above. EtOH administration at each time point significantly reduced the generation of ROS compared to samples that were stimulated but not treated with EtOH (Figure 7B). Since there is a direct link between the ROS production and the activity of the protein tyrosine phosphatase, as will be discussed later, the data support the idea that EtOH influenced the inflammasome activity via the protein tyrosine phosphatase.

## 3. Discussion

**Figure 8:**
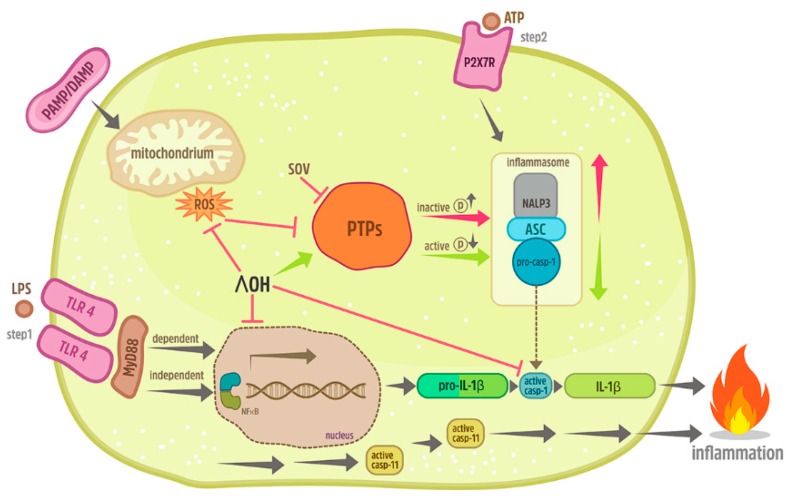
Mechanisms involved in influence of ethanol on inflammasome activation in human-derived liver cell line HepG2. Triggering the toll-like receptor (TLR)-4 with lipopolysaccharide (LPS) and/or the purinergic receptor P2X 7 (P2X7R) with adenosine triphosphate (ATP) induced the caspase-1 and apoptosis-associated speck-like protein containing caspase recruitment domain (CARD) (ACS), the key mediators of inflammasome activation. Co-stimulation with pathogen-associated or danger-associated molecular patterns (pathogen-associated molecular patterns (PAMP) or damage-associated molecular patterns (DAMP)) enhanced the production of reactive oxygen species (ROS). Treatment with sodium orthovanadate (SOV), the general inhibitor of tyrosine phosphatases (PTPs), led to caspase-1 activation, suggesting that high levels of phosphorylation of inflammasome components such as of ASC may play an important role in inflammasome regulation. Pre-incubation with MyD88 inhibitory peptide did not reduce the caspase-1 activation, suggesting that the LPS-mediated priming in HepG2 cells may also involve a MyD88-independent pathway. Exposure to acute ethanol reverses the LPS and ATP-induced activity of caspase-1 and ASC speck-like formation as well as the generation of ROS. Acute ethanol (EtOH) exposure may exert its inflammasome-suppressing impact via the loss of the ROS-mediated down-regulation of PTPs, in turn leading to the diminished activity of key mediators of the inflammasome assembly, such as ASC or caspase-1 (green arrows). Grey arrows indicate the inflammasome activation, while red arrows indicate its activation via PTPs.

Previous studies revealed significant anti-inflammatory effects of acute ethanol administration upon the induction of inflammatory processes in different cell entities including human lung epithelial cells [28,30], but also in hepatic and neuronal inflammation [17,31]. Despite its immense clinical relevance, the influence of an acute intoxication with ethanol on inflammatory processes mediated by inflammasomes is mechanistically poorly investigated. Since nothing is known about this interplay of hepatic cells, this study examined how ethanol, in such settings, will affect the molecular mechanisms in human HepG2 cells in vitro. We showed that activators of inflammasomes induced i) the formation of ASC specks and ii) the activation of caspase-1 in HepG2 cells. Moreover, the knock down of the key inflammasome factors has shown that mechanistical studies regarding inflammasome activation are applicable to HepG2 cells as well. After inflammasome activation, ASC assembles into a large protein complex, the ASC speck, which acts by bridging NLRP proteins, such as NLRP3, with procaspase-1 within the inflammasome complex, which subsequently results in the activation of caspase-1. Hence, ASC speck formation can be used as a simple upstream readout for inflammasome activation. Since the application of siRNA for caspase-1 results in a missing component for the ASC speck formation, the obtained results were expected. This is nicely confirmed by Figure 3 as well, which demonstrated that similar to a caspase-1 knockdown via siRNA, a down-regulation of NLRP3 by siRNA also reduced the speck formation. Hypothetically, if caspase-1 was available, ASC specks would be formed; yet, the activity of caspase-1 must not necessarily be changed, but it can be changed. Here was the expected difference in the application of the caspase-1 inhibitor. The ASC speck could be formed, however the caspase-1 inhibitor would, despite the speck formation, inhibit the caspase-1 activity. For these reasons, we have assessed both the speck formation as well as the caspase-1 activity in the underlying study. Furthermore, the purinergic P2X7 receptor seemed to be important for the activation of inflammasomes in HepG2 cells because the administration of the specific P2X7R antagonist decreased its activation, as shown by the reduced caspase-1 activity. Additionally, it decreased the tyrosine phosphatases activity, supporting the concept that the inflammasome activation in HepG2 cells was reversed upon the ethanol administration and thus indicating that the stimulation of the protein tyrosine phosphatase by ethanol may subsequently decrease the inflammasome activity (Figure 8).

Inflammasome activation is accompanied by pyroptosis, that is characterized by the activation of caspase-1 and the release of large amounts of functional oligomeric inflammasome particles containing both NLRP3 and ASC into the extracellular space to further potentiate the inflammation [2,22,32]. In our study, next to the stimulation with LPS and ATP, the cell viability was also significantly reduced by single stimulation with nigericin, a K^+^/H^+^ antiport ionophore (Figure 4b). Nigericin, which can be used as a positive control in pyroptosis experiments, destroys the cell integrity and thus decreases the cell viability, as observed in the present study. It is interesting that caspase-1 inhibition was not able to prevent this. Due to the time period between the stimulation with LPS and the administration of the caspase-1 inhibitor, it is likely that LPS itself induced an early apoptotic event, e.g., mediated through the autocrine secretion of TNF [33]. Thus, the caspase-1 inhibition may have prevented inflammasome-induced cell death, but not the inflammasome-independent LPS-induced cell death. Recently, it has been reported that LPS alone increased apoptotic cell death in HepG2 cells [34]. These findings underline the hypothesis about an inflammasome-independent LPS-reduced cell viability occurring in HepG2 cells. However, with regard to cell viability, in the underlying study the conclusions must be carefully interpreted since the applied assay detects living and not dead cells. The cytotoxicity can be estimated, but it does not necessary allow drawing conclusions about the real cell death. In further studies, other good apoptosis stains, like annexin V in combination with live/dead dyes to distinguish dead cells, live apoptotic cells and live cells, should be applied. While the same LPS concentration as was applied in our study was used, in contrast to our study design, the authors incubated HepG2 cells with LPS for 24 h. Furthermore, they observed a significant increase in the nuclear translocation of NF-κB p65 and ROS production upon LPS administration, which was similar to our results (Figure 5A and Figure 7b) [34]. Nonetheless, while the authors have provided further insights into the molecular mechanisms in human hepatoma cancer cells exposed to bacterial endotoxins, the impact of ethanol administration on inflammasomes, which will be discussed later, was not addressed. Besides the induction of NF-κB upregulation via the adaptor protein MyD88, LPS may upregulate NF-κB via the MyD88-independent pathway [35]. This possibly explains why the pre-incubation with the MyD88 inhibitor did not significantly reduce the LPS-induced NF-κB p65 phosphorylation (Figure 5A). A key regulator of the MyD88-independent pathway [36] was involved in licensing the NLRP3 inflammasome activation in the so-called non-canonical inflammasome pathway [37]. Since MyD88 inhibition neither affected the LPS and ATP nor LPS and nigericin-induced activation of caspase-1 (Figure 5B), the inflammasome activation in HepG2 cells may be related to both the canonical and the non-canonical pathway. This issue should be addressed in further studies in HepG2 cells.

Numerous studies describe the impact of ethanol administration on inflammasomes and their constituent elements [20,27]. Nonetheless, the role of inflammasomes in conditions of ethanol intoxication has mainly been studied in regard to chronic exposure, while here we concentrated on acute exposure. Chronic exposure to an ethanol-containing diet for a total period of five weeks induced high levels of NLRP3, ASC and caspase-1, resulting in increased inflammasome activity and thus enhanced inflammation in vivo [20,21]. In general, this setting is not comparable to our setting of an acute short-term exposure, which has been linked to the anti-inflammatory effects of ethanol [18]. Beside its capability of decreasing the pro-inflammatory cytokine levels in murine macrophages [38] or diminishing the adhesion of leukocytes to human lung epithelial cells [28], acute ethanol intake affects inflammasome activity. Upon acute ethanol administration, NLRP3 inflammasome activation was decreased by the inhibited oligomerization of ASC and lysosomal disruption [26]. In accordance with these studies, the caspase-1 activity and ASC speck formation were diminished in our study upon ethanol administration as well (Figure 6A–F and Figure 7A). Despite comparable findings, in contrast to our study, Nurmi et al. primed primary human macrophages with LPS (1 ug/mL) for 3 h then pre-incubated the cells with varying concentrations of ethanol before inflammasome activators were added. While they revealed a dose-dependent decrease in IL-1β production, reduced caspase-1 activation and the diminished oligomerization of ASC, we applied ethanol in only one dose. It remains to be further evaluated if HepG2 cells have a dose dependency, as was observed in human macrophages. We assessed the levels of IL-1β in cell culture supernatants, however the data must be carefully interpreted since the dilution factor applied by the used cell culture medium was too high The IL-1β level, upon LPS and ATP stimulation, was significantly higher compared with the unstimulated controls (5.15 ± 1.79 vs. 0.01 ± 0.00 pg/mL, *p* < 0.05). Ethanol application significantly reduced the IL-1β levels compared to the LPS and ATP-stimulated controls (E1: 0.76 ± 0.35 and E2: 0.47 ± 0.22 vs. 5.15 ± 1.79 pg/mL, both *p* < 0.05). Yet, enhanced IL-1β, upon LPS and ATP stimulation, confirmed the findings of the present study that LPS and ATP induce inflammasome and caspase-1 activation. 

The phosphorylation and dephosphorylation of inflammasome components NLRP3 and ASC play an important role in inflammasome activation [39,40,41]. ASC phosphorylation acts as a molecular switch that controls the formation of speck-like aggregates and inflammasome activity [39]. Consistent with these studies, an increased caspase-1 activity after treatment with the general inhibitor of protein phosphotyrosyl phosphatases was observed (Figure 7a), indicating that a decreased activity of the tyrosine phosphatases may support inflammasome activation in HepG2 cells. However, ethanol administration diminished the caspase-1 activity and inhibited the ASC speck formation (Figure 6A–F and Figure 7A). In brain neurons of rats, a short-term exposure to ethanol led to the tyrosine phosphatase-mediated dephosphorylation of N-methyl-daspartate receptor (NMDAR) subunits [42]. Recently, Hoyt et al. reported that acute ethanol administration dose dependently—as discussed before—inhibited the activation of the NLRP3 inflammasome, addressing the impact of alcohol on the stimulation of the protein tyrosine phosphatase [27]. Interestingly, ROS were capable of inhibiting protein-tyrosine phosphatases, resulting in an elevated intracellular phosphorylation level [43]. Hoyt et al. revealed a significant decrease in ROS generation after short-term ethanol administration in the LPS and ATP-stimulated PBMC [27]. Consistent with the findings in macrophages and monocyte-derived dendritic cells [43,44], the stimulation with LPS and/or ATP significantly increased the ROS generation in HepG2 cells as well. Since the stimulation periods (2 or 48 h vs. 5 h) as well as the LPS concentration (100 ng/mL vs. 1 μg/mL) were substantially different in those studies compared with our study, the induced mechanisms regarding the ROS formation may not be crucially sensitive to the dose or timing issues. In summary, ethanol may exert its inflammasome-suppressing impact via diminishing the ROS generation, in turn leading to a loss of the ROS-mediated tyrosine phosphatases inhibition. 

It has been shown that ethanol blocks the NLRP3 inflammasome activation in several cell types, including human peripheral blood mononuclear cells, murine bone marrow-derived dendritic cells and neutrophils. Our study extends the knowledge on molecular pathways and reveals potential key mediators of the inflammasome function upon acute ethanol administration in HepG2 cells. Yet, to exclude the notion that our findings are specific to HepG2, further studies with different cell entities are necessary. Inflammasome components are present in multiple cell types, including HepG2 cells (Figure 3) [45,46]. The rationale for using HepG2, which is most commonly used in drug metabolism and hepatotoxicity studies [47], was determined by the fact that our and others’ previous studies have already revealed the significant anti-inflammatory effects of acute alcohol administration upon the induction of inflammatory processes in various cell entities, including lung epithelial cells [28,29,30,48], human endothelial cells [49], macrophages and others [38,50]. Though the main mechanisms underlying the anti-inflammatory potential of acute ethanol intoxication under inflammatory conditions were linked to NF-κB [17], the impact of acute ethanol application on inflammatory processes specifically in terms of inflammasome functionality is mechanistically poorly investigated in hepatic cells. However, the experimentally narrow scope of this study leaves some questions open at the moment and further studies are necessary. Moreover, modifying the treatment strategies by applying other DAMP and PAMP will deliver further insights into the understanding of the molecular pathways of inflammasome activation. Additionally, the specific influence of ROS on inflammasomes should be analyzed by applying ROS scavengers. In addition, optimizing the cell culture to allow a better and more reliable IL-1β detection range should be considered in future experimental settings. In addition, the effects of prolonged exposure to ethanol as well as another dose of ethanol to assess potential dose dependency should be analyzed. Additionally, the imaging techniques should be applied to visualize the translocation of NF-κB into the nucleus instead of only providing the levels of its activity.

## 4. Materials and Methods 

### 4.1. Reagents

The lipopolysaccharide (LPS; from E. coli O26:B6), BzATP (ATP), nigericin, sodium orthovanadate, AC-YVAD-CMK and ethanol were all purchased from Sigma-Aldrich (Steinheim, Germany). The Pepinh-MYD (MyD88 inhibitory peptide, PI MyD88) was purchased from Invivogen (San Diego, CA, USA). The AZ10606120 dihydrochloride was acquired from Tocris Bioscience (Bristol, United Kingdom). The PYCARD (Apoptosis-associated speck-like protein containing a CARD) polyclonal antibody and Cellrox Green Reagent were obtained from Thermo Fisher Scientific (Waltham, MA, USA). The Caspase-Glo^®^ 1 Inflammasome Assay was purchased from Promega (Madison, WI, USA). The Calcein AM Cell Viability Assay was purchased from R&D Systems (Wiesbaden, Germany).

### 4.2. Cell Culture

The HepG2 cell line (Cell Line Services, Heidelberg, Germany) was cultured at 37°C under 5% CO_2_ in an RPMI-1640 medium (Seromed, Berlin, Germany) supplemented with 10% heat-inactivated fetal calf serum (FCS), 100 IU/mL penicillin, 100 µg/mL streptomycin (Gibco, Karlsruhe, Germany) and 20 mM 4-(2-hydroxyethyl)-1-piperazineethanesulfonic acid (HEPES) buffer (Sigma, Steinheim, Germany). The culture media were replaced each second or third day and the cells were not used beyond passage 20 to maintain equal conditions between the experiments.

### 4.3. Stimulation Protocol

The HepG2 cells were plated at 100,000 cells per well in 96-well plates. After growing overnight, the medium was replaced by fresh medium. To investigate the general principles of inflammasome activation in HepG2 cells, the cells were primed with LPS (1 µg/mL, step 1) for five hours and then treated with BzATP (100 µM) or nigericin (50 µM, step 2) for one hour. In order to study whether the MyD88-dependent or independent pathway is involved in TLR4 activation by LPS, after the replacement of the medium, the cells were pre-incubated with MyD88 inhibitory peptide (25 µM) for one hour and then treated as described.

To further examine whether distinct mediators contribute to the activation of inflammasome in HepG2 cells, diverse inhibitors like sodium orthovanadate (general inhibitor of tyrosine phosphatases, 1000 µM), AC-YVAD-CMK (inhibitor of caspase-1, 100 µM) or AZ10606120 (inhibitor of purinergic receptor P2X7R, I P2X7, 1 µM) were added four hours after LPS priming (one hour before the administration of ATP or nigericin).

To study the influence of ethanol on inflammasome activation, the cells were treated with ethanol (170 mM corresponding to one vol vol^−1^ per cent or 7.9 mg EtOH ml^−1^) at two time points. The first time point was simultaneous with the LPS application (EtOH 1); the second time point was simultaneous with applying the ATP or nigericin (EtOH 2) or corresponding controls.

Finally, the cellular supernatants and lysates were collected for further analyses. The corresponding schematic timeline of the experimental design is shown in Figure 1.

The concentrations of the reagents and of EtOH were based on previous work to allow a better comparison of data [27,28,51].

### 4.4. Immune Cytological Staining of SPECKs

In order to determine the SPECK formation, the HepG2 cells were transferred to 8-well chamber slides (100,000 cells per well, Nunc^®^ Lab-Tek^®^ II Chambered Coverglass, Sigma-Aldrich) and allowed to grow overnight. In order to investigate the time-dependent and mechanistical effects of ethanol on inflammasome activation, ethanol was applied at two different time points (EtOH1 as shown in Figure 1A and EtOH2 as shown in Figure 1B). Briefly, the cells of the EtOH1 group (Figure 1A) were simultaneously treated with LPS and EtOH for 5 h before the ATP was added. In contrast, the cells of the EtOH2 group (Figure 1B) were primed with LPS for 5 h and then EtOH was added together with ATP. After fixation of the cells with ice-cold 4% paraformaldehyde for 10 min and subsequent washing procedures, the cells were permeabilized with 0.5% Triton X-100 solution for 5 min at room temperature and washed. Skim milk as a blocking buffer (10%) was applied for 45–60 min at room temperature for background reduction. After washing, the primary ASC antibody (PYCARD Polyclonal AB; Thermo Fisher Scientific, Waltham, MA, USA) was added in a final concentration of 5 µg/mL for an incubation period of one hour at room temperature (dilution 1:100). Subsequently, the primary antibody was washed away and the secondary antibody (Alexa Fluor 488; Invitrogen, San Diego, CA, USA) was added for one hour at room temperature (dilution 1:1000). Finally, the cells were washed and the nuclei were stained with 4′,6-Diamidin-2-phenylindol (DAPI) which was already provided in the mounting medium (VECTASHIELD Antifade Mounting Medium with DAPI; VECTOR Laboratories, Burlingame, CA, USA). The mean fluorescence intensity of the ASC specks was assessed in five randomly chosen different fields per sample by using the Zeiss Axio Observer Z1 fluorescence microscope (x10 objective) (from Zeiss, Gottingen, Germany).

### 4.5. Knock Down of Inflammasome Components Via Small Interfering (si)RNA

Forty thousand HepG2 cells per well were transferred to 8-well chamber slides. The cells were transfected with siRNA directed against caspase-1 (CASP1 siRNA, 25 pmol, Entrez Gene ID834, Thermo Fisher Scientific, Germany), NLRP3 (NLRP3 siRNA, 25 pmol, Entrez Gene ID114548, Thermo Fisher Scientific, Germering, Germany) or with the control siRNA (Silencer™ Cy™3-labeled Negative Control No. 1 siRNA, 25 pmol, Thermo Fisher Scientific, Germany), by using Lipofectamine^®^ RNAiMAX Reagent according to the manufacturer‘s instructions for the Lipofectamine^®^ RNAiMAX Reagent transfection procedure (Invitrogen by Life technologies). The nontreated cells and cells transfected with the control siRNA (negative control, NC) served as controls. The transfection efficiency was evaluated by the quantification of positively transfected cells (Cy3-positive cells). After transfection of the cells for 24 h, they were exposed to LPS and ATP, as indicated above.

### 4.6. Caspase-1 Activity Assay

Caspase-Glo^®^ 1 Inflammasome Assay (Promega, Madison, WI, USA) was used to detect activated caspase-1 in the cultured cells. One hundred thousand cells per well were seeded into clear-bottom, white opaque 96-well microplates (Sigma Aldrich, Steinheim, Germany) and treated as described above. The Caspase-Glo^®^ 1 Reagent was prepared and added to the wells of the 96-well plate according to the manufacturer’s instructions. Then, the cells were incubated for one and a half hours and the luminescence was measured by Tecan Microplate Reader (Tecean Group, Männedorf, Switzerland) using the software Magellan.

### 4.7. Cell Viability

The cell viability was assessed using CalceinAM Cell Viability Assay (R&D Systems, Minneapolis, MN, USA). As a non-fluorescent, hydrophobic compound, CalceinAM easily permeates intact, live cells. The hydrolysis of CalceinAM by intracellular esterases produces calcein, a hydrophilic, strongly fluorescent compound that is well-retained in the cell cytoplasm. A total of 100,000 cells per well were plated in clear bottom, black-walled 96-well plates (BD Biosciences, Franklin Lakes, NJ, USA) and treated according to the stimulation protocol. The CalceinAM Working Solution was prepared according to the manufacturer’s instructions. After removing the media, the cells were washed twice with CalceinAM DW Buffer (100 µL and 50 µL). Then, the CalceinAM Working Solution (50 µL) was added to the wells and incubated for 30 min. The fluorescence intensity was recorded by a Twinkle LB 970 Microplate Fluorometer using a 490 nm excitation filter and a 520 nm emission filter (software MikroWin 2000). The viability was calculated, since the fluorescence intensity is proportional to the number of viable cells.

### 4.8. Determination of the NF-κB Activity

The HepG2 cells were plated in 24-well plates at 500,000 cells per well and treated according to the stimulation protocol. For the analysis of the NF-κB p65 subunit, the cells were lysed and processed according to the manufacturer’s instructions using the PathScan^®^ Phospho-NF-κB p65 (Ser536) Sandwich ELISA Kit (Cell Signaling Technology: Danvers, MA, USA). Briefly, the cell lysates (100 µg protein) were prepared according to the manufacturer´s instructions. Subsequently, 100 µL of cell lysates was added to each well and incubated for two hours at 37 °C. After the incubation period, the wells were washed four times with 200 µL 1× Wash Buffer. Then, 100 μL of the reconstituted detection antibody was added and incubated at 37 °C for one hour. Subsequently, the washing procedure was repeated and 100 µL of reconstituted horseradish peroxidase-conjugated secondary antibody was added to each well. After thirty minutes, the wells were washed again and 100 µL of the chemiluminescent substrate was added and the luminescence was captured after ten minutes, as suggested by the manufacturer. The percentage of the active NF-κB p65 related to the control was calculated.

### 4.9. Measurement of the Intracellular ROS

The levels of intracellular reactive oxygen species (ROS) were detected using CellROX™ Green Reagent (Thermo Fisher Scientific, Waltham, MA, USA). One hundred thousand HepG2 cells per well were transferred to clear bottom, black-walled 96-well plates and treated as described before. Subsequently, the CellROX^®^ Reagent (ready-to-use, final concentration of 5 μM) was added and incubated for 30 min according to manufacturer’s suggestions. Then, the medium was removed and the cells were washed three times with PBS. The fluorescence signal was determined using a Twinkle LB 970 Microplate Fluorometer (extinction/emission ratio 485/520 nm).

### 4.10. Statistical Analysis

A GraphPad Prism 6 (GraphPad Software Inc., San Diego, CA, USA) was used to perform the statistical analyses. The normality of all the data was analyzed by the Kolmogorov–Smirnov test. Differences between the groups were determined by a one-way analysis of variance (ANOVA) with a Dunn post-hoc test for comparison among all the different groups. A *p*-value of below 0.05 was considered significant. Data were given as mean ± the standard error of the mean (s.e.m.). All the experiments were performed five times.
